# The prevalence and burden of subthreshold generalized anxiety disorder: a systematic review

**DOI:** 10.1186/1471-244X-14-128

**Published:** 2014-05-01

**Authors:** Heidemarie Haller, Holger Cramer, Romy Lauche, Florian Gass, Gustav J Dobos

**Affiliations:** 1 Department of Internal and Integrative Medicine, Kliniken Essen-Mitte, Faculty of Medicine, University Duisburg-Essen, Am Deimelsberg 34a, 45276 Essen, Germany

**Keywords:** Anxiety disorders, Epidemiology, Burden of illness, Systematic review

## Abstract

**Background:**

To review the prevalence and impact of generalized anxiety disorder (GAD) below the diagnostic threshold and explore its treatment needs in times of scarce healthcare resources.

**Methods:**

A systematic literature search was conducted until January 2013 using PUBMED/MEDLINE, PSYCINFO, EMBASE and reference lists to identify epidemiological studies of subthreshold GAD, i.e. GAD symptoms that do not reach the current thresholds of DSM-III-R, DSM-IV or ICD-10. Quality of all included studies was assessed and median prevalences of subthreshold GAD were calculated for different subpopulations.

**Results:**

Inclusion criteria led to 15 high-quality and 3 low-quality epidemiological studies with a total of 48,214 participants being reviewed. Whilst GAD proved to be a common mental health disorder, the prevalence for subthreshold GAD was twice that for the full syndrome. Subthreshold GAD is typically persistent, causing considerably more suffering and impairment in psychosocial and work functioning, benzodiazepine and primary health care use, than in non-anxious individuals. Subthreshold GAD can also increase the risk of onset and worsen the course of a range of comorbid mental health, pain and somatic disorders; further increasing costs. Results are robust against bias due to low study quality.

**Conclusions:**

Subthreshold GAD is a common, recurrent and impairing disease with verifiable morbidity that claims significant healthcare resources. As such, it should receive additional research and clinical attention.

## Background

Mental disorders and anxiety disorders in particular seem to continually increase in incidence and prevalence [1-3], raising questions about the early detection of potential risk factors and the nature of ‘subthreshold’ states. Individuals in such states experience psychopathological symptoms that are mild, atypical, masked and/or brief but recurrent; which however fail to reach the Diagnostic and Statistical Manual of Mental Disorders (DSM) [4] or World Health Organization’s International Classification of Diseases (ICD) [5] standardized diagnostic thresholds by reason of their number and/or duration. Whilst these states often cause significant suffering and impairment [6-9], their doubtful morbidities and lack of standardized definition lead to ongoing marginalization [10]. Diagnostic thresholds current disregard for subthreshold, subsyndromal and subclinical symptoms may lead to considerable false negative cases. For example, a 2007 American study of 5692 adult mental health care users found that only 61.2% had a DSM-IV diagnosis; a non-negligible percentage of mental health service, however, provided to patients suffering from subthreshold emotional problems [11].

Whilst previous systematic research has revealed consistent evidence of the impact of various subthreshold mental disorders [12-18], past studies of generalized anxiety disorder (GAD) below the current diagnostic thresholds have varied widely in their quality and results, with only non-systematic reviews having been conducted to date [19]. To determine the morbidity of subthreshold GAD, representative epidemiological data are required. The current review therefore aimed to systematically assess prevalence, chronicity risk, human and economic burden as well as treatment need of subthreshold GAD.

## Methods

The review was planned and conducted in accordance with the MOOSE (Meta-Analysis Of Observational Studies in Epidemiology) [20] guidelines.

### Literature search strategy

A systematic literature search was undertaken using both electronic and manual methods. The former entailed searching PUBMED/MEDLINE, PSYCHINFO and EMBASE databases, from their inception until January 2013. The search strategy focused on terms for ‘subthreshold’ and ‘anxiety’, with ‘epidemiology’, ‘human and economic burden’ and ‘prevention and therapy’. The precise methods used were tailored to each database in turn. The full search strategy for the PUBMED was: *(subthreshold [Title/Abstract] OR subsyndromal [Title/Abstract] OR subclinical [Title/Abstract]) AND anxiety [Title/Abstract] AND (Anxiety Disorders/epidemiology [MeSH Terms] OR anxiety disorders/economics [MeSH Terms] OR anxiety disorders/complications [MeSH Terms] OR disability [Title/Abstract] OR impairment [Title/Abstract] OR cost of illness [MeSH Terms] OR health care costs [MeSH Terms] OR comorbidity [MeSH Terms] OR anxiety disorders/therapy [MeSH Terms] OR anxiety disorders/control and prevention [MeSH Terms])*. No restrictions were placed on language, article type or year of publication. Identified papers’ references were screened manually to find additional related studies.

### Eligibility criteria

Having read the located abstracts, researchers went on to assess eligibility of selected full-text articles. Eligible articles were required to meet the following criteria:

– Type of study: Only epidemiological trials with original data, published in English or German as full articles in peer-reviewed journals were included. Reviews, study protocols, expert statements, diagnostic or methodological pieces were all excluded.

– Diagnostic criteria: Only studies that defined subthreshold GAD by relaxing one or more of the diagnostic criteria from the standardized diagnostic manuals (DSM-III-R, DSM-IV or ICD-10) were included. Papers that used earlier versions of the DSM or ICD were excluded, because of widely deviating threshold definitions for GAD.

– Study samples: Only studies representative of the general population (including sub-populations of several age groups), or of specific patient populations (e.g. primary care or psychiatry), were included. Studies that treated subthreshold GAD as a comorbid symptom of other physical or mental disorders were excluded. Where two or more articles reported data from the same study sample, only the most relevant article was considered.

### Data extraction

Studies’ characteristics were extracted independently by two reviewers. Inconsistencies were rechecked within the research team and resolved by discussion. The following data were extracted: setting (specific patient population, general population, adolescents, older adults), country, study period of data collection, study design (longitudinal, cross-sectional, retrospective), sample size, age range, assessment of GAD (type of interview, questionnaire), definition of subthreshold GAD used and main findings of the study.

### Assessment of methodical quality

A scoring system previously used for observational trials [21] was adapted to critically appraise the located epidemiological studies, awarding one point for each of the following criteria:

1. Random population sample with unbiased sampling strategy

2. Adequate sample size (>1000)

3. Adequate response rate (>70%)

4. Comparison between respondents/non-respondents (those who refuse the initial query)

5. Reliable and valid assessment of GAD (standardized instruments used).

Studies were rated as ‘low quality’, if they scored less than three on five points, and as ‘high quality’ from three to five points. The quality assessment was undertaken by two reviewers independently, with comparison, discussion and agreement by consensus.

### Data analysis

The median prevalence of subthreshold GAD was calculated for each homogeneous study population. Sensitivity analyses of low versus high quality studies’ scores were then performed, to test the robustness of studies’ outcome data. Additional study outcomes were summarized qualitatively.

## Results

### Search results

**Figure 1:**
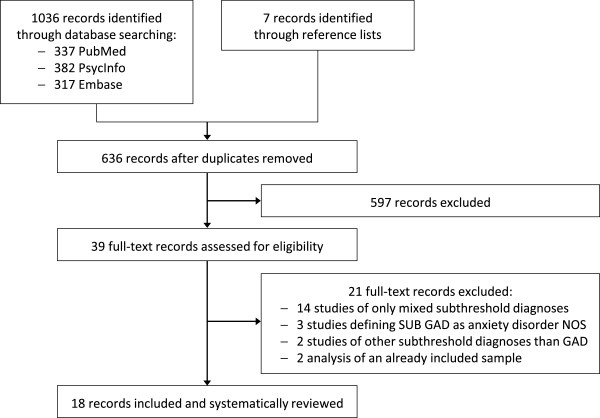
Flowchart of study exclusions.

Electronic database searching identified 1036 papers. Another seven emerged from a manual search of studies’ reference lists. Of this total of 1043 papers, 407 proved to be duplicates. The remaining 636 abstracts were then screened, with 39 studies being read in full-text to assess their eligibility for inclusion in the review. Of these, 21 studies [22-42] were excluded because they reported outcome data only for mixed subthreshold diagnoses, for subthreshold diagnoses other than GAD or because they contained data analyzed in already included articles. Other studies were excluded because they defined subthreshold GAD as an anxiety disorder not otherwise specified, also comprising subjects with various anxiety and/or depressive symptoms that did not clearly match a single diagnostic category. Eighteen epidemiological studies were finally included and reviewed [43-60] (Figure 1).

### Study characteristics

The reviewed studies were conducted between 1979 and 2006 (Table 1). Most were cross-sectional in nature, with the exception of two studies [43,52], and were based on European [43,44,46,48,49,55,57,58,60] or North American [45,47,50,51,53,54,56,59] data. One cross-cultural study was included [52]. A total of 62,501 participants between 15 and 96 years was enrolled in these studies, but data on only 48,241 of this number remained when different studies of the same sample were subtracted [44,46,47,51,52,54,56,58]). Participants came from the general population [43-46,51,56], were older adults [47,48,54,59], adolescents [49] or primary care patients [50,52,53,55,57,58,60].

**Table 1:** Description of included studies

**Source**	**Setting**	**Study period**	**Study design**	**Sample size (n)**	**Age range**	**Assessment of GAD**	**Definition of subthreshold GAD**	**Main findings**
Angst, 2006 [43]	General population (Switzerland | ZCS)	1979-1999	Longitudinal	591	19–41	Interview (SPIKE)	DSM-III-R diagnosis of GAD with relaxed duration criterion (1 versus 3 months of duration)	- Point prevalence of SUB GAD = 6.2% for 3-month GAD/7.7% for 1-month GAD
								- High levels of distress, social and work impairment & comparable comorbidity rates and suicide attempts in all SUB GAD groups (sign. differences compared to controls, but no sign. differences compared to 6-month GAD)
								- Same age of onset, course, and treatment rates in SUB GAD and GAD cases
								- 57.5% of treated patients had SUB DSM-III-R GAD & 50% had SUB DSM-IV GAD
Beesdo, 2009 [44]	General population (Germany | GHS)	1998-1999	Cross-sectional	4181	18–65	Interview (CIDI)	Anxious worrying for at least 3 months with at least 2 of the other DSM-IV criteria for GAD	- Higher associations between GAD, also on the SUB level, and medically unexplained pain compared to other anxiety disorders (independent from comorbid depression)
								- Lowest quality of life, greatest disability and health care utilization in the group with both unexplained pain and (SUB) GAD
Bienvenu, 1998 [45]	General population (United States | ECA)	1993	Cross-sectional	1920	≥27	Interview (DIS)	Group 1: DSM-III-R GAD with duration of 1–6 month / Group 2: DSM-III-R GAD of 1–6 month + fewer than 6 associated symptoms	- Lifetime prevalence of SUB GAD = 8% for group 1/12% for group 2
								- Higher SUB GAD prevalence in women and younger adults
Carter, 2001 [46]	General population (Germany | GHS)	1998-1999	Cross-sectional	4181	18–65	Interview (CIDI)	Persistent worrying for at least 3 months with at least 2 of the other DSM-IV criteria for GAD	- 12-month prevalence of SUB GAD = 2.1%
								- Higher prevalence in women and in older adults
								- High levels of distress and impairment in younger and older SUB GAD cases
								- Same comorbidity rates in SUB GAD and threshold GAD (commonly occurring: other anxiety disorders, depression, and somatoform disorders)
Grenier, 2011 [47]	Community-dwelling older adults (Canada|ESA)	2005-2006	Cross-sectional	2784	≥65	Interview (ESA-Q)	Symptoms of anxiety, not meeting all symptom criteria of DSM-IV GAD	- 12-month prevalence of SUB GAD = 3.0%
								- Chronic physical health problems, social disability, use of benzodiazepines and comorbid depression not sign. different between SUB GAD and threshold GAD, but sign. higher in SUB GAD than in controls
Heun, 2000 [48]	Older adults (Germany)	1993-1994	Cross-sectional	287	≥60	Interview (CIDI)	DSM-II-R GAD of 6 months, but fewer than 6 associated symptoms	- Lifetime prevalence of SUB GAD = 5.2%
								- Higher SUB GAD prevalence in women
Hoyer, 2002 [49]	Young women, (Germany|DPS)	1996-1997	Cross-sectional	2064	18–25	Interview (ADIS)	Fulfilling 3 out of 4 DSM-IV criteria for GAD	- Point prevalence of SUB GAD = 2.4%
								- 42% of the SUB GAD cases have other comorbid mental disorders
								- Sign. reduced psychosocial functioning in SUB GAD cases compared to controls
Kertz, 2011 [50]	Primary care patients (United States)	NR	Cross-sectional	329	22–88	Interview (PRIME-MD)	GAD symptoms fulfilling DSM-IV criterion A in addition to 1 or 2 of the other GAD criteria	- Point prevalence of SUB GAD = 6%
								- Point prevalence of GAD symptoms = 24%
								- SUB GAD as risk factor for threshold GAD
								- Sign. poorer physical health, greater stress and sleep difficulty in SUB GAD than in the no worry group; but no sign. differences between GAD, SUB GAD and no worry group on health care utilization and work productivity
Kessler, 2005 [51]	General population (United States|NCS-R)	2001-2003	Cross-sectional	9282	≥18	Interview (CIDI, SCID)	DSM-IV diagnosis of GAD with relaxed duration criterion (1 versus 3 months of duration)	- Point prevalence of SUB GAD = 2.1% for 3-month GAD / 2.6% for 1-month GAD
								- 12-month prevalence of SUB GAD = 3.9% for 3-month GAD / 5.5% for 1-month GAD
								- Lifetime prevalence of SUB GAD = 8% for 3-month GAD / 12.7% for 1-month GAD
								- Onset, persistence, comorbidity, social and work impairment not greatly different between 1–5 months GAD and over 6 months GAD
								- Short SUB GAD episodes typically recur over years
Maier, 2000 [52]	Primary care patients (Cross-cultural|PPGHC)	1991-1992	Longitudinal	5604	15–65	Interview (CIDI)	All ICD-10 GAD symptom criteria, but relaxed time criterion (<1 versus 1–6 months of duration)	- Not sign. smaller psychosocial disability for SUB GAD with 1–6 months (24.3%) and with GAD over 6 month (24.9%), a little smaller in <1 month SUB GAD (17.3%), higher than in those with chronic somatic diseases (19.5%)
								- Higher disability in (SUB) GAD with other comorbid psychiatric syndromes
Olfson, 1996 [53]	Primary care patients (United States|SDDS-PC)	1994	Cross-sectional	1001	35–65	Interview (SCID)	Excessive anxiety for the past 6 months, not meeting full DSM-III-R criteria for GAD	- Point prevalence of SUB GAD = 6.6%
								- Higher prevalence in younger adults
								- 48.5% met criteria for another mental disorder
								- After adjustment for covariates, no more differences on work, family, social function, and health care utilization in SUB GAD compared to controls
Potvin, 2011 [54]	Community-dwelling older adults (Canada|ESA)	2005-2006	Cross-sectional	2414	65–96	Interview (ESA-Q)	At least 1 essential symptom of a DSM-IV GAD without fulfilling all criteria	- In men, global cognitive impairment is sign. linked to SUB GAD whether depression was comorbid or not
Rucci, 2003 [55]	Primary care patients (Italy|PPGHC + BS)	1991-1992	Cross-sectional	554	15–65	Interview (CIDI)	3+ ICD-10 GAD criteria of 1-month duration including apprehension, motor tension, and automatic overactivity	- Point prevalence of SUB GAD = 8.3%
								- Higher SUB GAD prevalence in women
								- (SUB) GAD and depression were the most frequent disorders
								- SUB GAD as a precursor of threshold GAD
								- Poorer health perception and higher psychological distress in SUB GAD than in controls, but no sign. differences in physical disability
Ruscio, 2007 [56]	General population (United States|NCS-R)	2001-2003	Cross-sectional	5692	≥18	Interview (CIDI, SCID)	DSM-IV symptom criteria for GAD, relaxed duration of 1+ months, also non-excessive worry, and only 2+ criterion C symptoms	- 12-month prevalence of SUB GAD = 6.6%
								- Lifetime prevalence of SUB GAD = 13.7%
								- Risk of comorbid psychiatric disorders equal for GAD (92.1%) and SUB GAD (86.3%)
								- Sign. risk of onset of various comorbid anxiety & mood disorders caused by SUB GAD
Szadoczky, 2004 [57]	Primary care patients (Hungary)	1998-1999	Cross-sectional	1815	18–65	Interview (DIS)	1 to 5 symptoms of DSM-III-R GAD and duration of less than 6 months	- Point prevalence of SUB GAD = 5.7%
								- 12-month prevalence of SUB GAD = 10.9%
								- Higher SUB GAD prevalence in women
Weiller, 1998 [58]	Primary care patients (Europe|PPGHC)	1991-1992	Cross-sectional	1973	≤65	Interview (CIDI)	4+ symptoms of ICD-10 GAD with 1 automatic arousal symptom, 3–6 months or all ICD-10 criteria, but no automatic arousal symptom or all ICD-10 criteria, but <4 symptoms	- Point prevalence of SUB GAD = 4.1%
								- Sign. poorer overall health status and higher psychosocial disability in SUB GAD patients than in controls, and no differences between GAD and SUB GAD
								- Sign. more general practitioner visits for psychological problems in SUB GAD than controls (but no more anxiolytics or antidepressants in adjusted statistics)
								- 39% of SUB GAD an 33% of GAD cases were identified as clinical relevant
Wetherell, 2003 [59]	Older adults (United States)	NR	Cross-sectional	90	55–88	Interview (ADIS)	Anxiety symptoms, not meeting criteria for DSM-IV GAD	- Sign. more sleep disturbance, fatigue, distress/impairment, higher history of psychotherapy, history and current medication use (antidepressants + benzodiazepines) in GAD and SUB GAD than in controls
								- Current psychotropic medication use in 54.5% of SUB GAD (vs. 6.3% in controls)
Wittchen, 2002 [60]	Primary care patients (Germany)	2000	Cross-sectional	17739	≥16	Questionnaire (GAS-Q)	Full DSM-IV GAD, but of 1–6 months of duration	- Point prevalence of SUB GAD = 1.3%
								- Point prevalence of GAD symptoms = 21.7%
								- Higher point prevalence in women
								- No sign. differences between SUB GAD and GAD on onset, course, and disability

*Abbreviations*: *ADIS* Anxiety Disorder Interview Schedule, *BS* Bologna Study, *CI* Confidence Interval, *CIDI* Composite International Diagnostic Interview, *DIS* Diagnostic Interview Schedule for DSM, *DPS* Dresden Predictor Study, *DSM* Diagnostic and Statistical Manual of Mental Disorders, *ECA* Epidemiologic Catchment Area Program, *ESA* Enquête sur la Santé des Aînés Study, *GAD* Generalized Anxiety Disorder, *GAS-Q* Generalized anxiety screening questionnaire, *GHS* German National Health Interview and Examination Survey, *ICD* International Statistical Classification of Diseases and Related Health Problems, *n* Number of Study Participants, *NCS-R* National Comorbidity Survey Replication, *NR* Not Reported, *PPGHC* WHO International Study on Psychological Problems in General Health Care, *PRIME-MD* Primary Care Evaluation of Mental Disorders Structured Psychiatric Interview, *SCAN* Schedules for Clinical Assessment in Neuropsychiatry, *SCID* Structured Clinical Interview for DSM Diagnoses, *SDDS-PC* Symptom-Driven Diagnostic System for Primary Care, *sign* significant, *SPIKE* Structured Psychopathological Interview and Rating of Social Consequences of Psychic Disturbances for Epidemiology, *SUB* Subthreshold, Subclinical, or Subsyndromal, *ZCS* Zurich Cohort Study.

### Study quality

Fifteen of the reviewed studies were assessed as being of high quality [43-47,49,51-58,60] and three of low quality [48,50,59] (Table 2). All but one of the former [55] used unbiased random population samples greater than 1000, with eight studies reporting response rates greater than 70% [44,46,51,52,54,56,57,60]. The low quality studies did not fulfill any of these criteria. Twelve of the reviewed studies performed analyses of non-respondents [43-46,48,50,51,53-56,60]. All but one study [60] used standardized interviews to assess study diagnosis for subthreshold GAD.

**Table 2:** Quality assessment of epidemiological studies included

**Criterion**	**Unbiased random population sample**	**Adequate sample size (>1000)**	**Adequate response rate (>70%)**	**Analysis of non-respondents**	**Reliable and valid assessment of GAD**	**Total quality score (max. 5)**

Angst, 2006 [43]	1	1^a^	0^b^	1^b^	1	4
Beesdo, 2009 [44]	1	1	1	1^e^	1	5
Bienvenu, 1998 [45]	1	1	0	1^c^	1	4
Carter, 2001 [46]	1	1	1	1	1	5
Grenier, 2011 [47]	1	1	0	0	1	3
Heun, 2000 [48]	0	0	0	1^d^	1	2
Hoyer, 2002 [49]	1	1	0	0	1	3
Kertz, 2011 [50]	0	0	0	1	1	2
Kessler, 2005 [51]	1	1	1	1^f^	1	5
Maier, 2000 [52]	1^g^	1^g^	1^g^	0^g^	1	4
Olfson, 1996 [53]	1	1	0	1	1	4
Potvin, 2011 [54]	1	1	1	1	1	5
Rucci, 2003 [55]	1	0	0	1	1	3
Ruscio, 2007 [56]	1	1	1	1^f^	1	5
Szadoczky, 2004 [57]	1	1	1	0	1	4
Weiller, 1998 [58]	1	1	0	0	1	3
Wetherell, 2003 [59]	0	0	0	0	1	1
Wittchen, 2002 [60]	1	1	1	1	0	4

^a^the stratified sample represents 2600 persons; ^b^from [61]; ^c^from [62]; ^d^from [63]; ^e^from [64]; ^f^from [65]; ^g^from [66].

### Study results

#### Prevalence estimates

Median prevalence estimates were conducted for the different study populations (Table 3) and definitions of subthreshold GAD (Table 4). Two studies [43,51] found point prevalence rates for subthreshold GAD amongst adults in the general population (median 4.4%, range 2.1% - 7.7%). Four others [46,51,56,57] reported 12-month prevalence rates with a median of 3.9% (range 2.1% - 6.6%) and/or lifetime prevalence rates with a median of 12% (range 8% - 13.7%) [45,51,56] for the general population.

**Table 3:** Median prevalence rates of mixed subthreshold GAD diagnoses

	**Point prevalence**	**12-month prevalence**	**Lifetime prevalence**
General population	4.4% (N = 2)	3.9% (N = 4)	12% (N = 3)
Adolescents	2.4% (N = 1)	n/a	n/a
Older adults	n/a	3% (N = 1)	5.2% (N = 1)
Primary care patients	5.9% (N = 6)	10.9% (N = 1)	n/a

*Abbreviations*: *N* Number of Studies, *n/a* not available.

**Table 4:** Median prevalence rates of 1-month and 3-month subthreshold GAD for the general population

	**Point prevalence**	**12-month prevalence**	**Lifetime prevalence**
SUB GAD of >1-month duration	5.2% (N = 2)	6.1% (N = 2)	12.4% (N = 3)
SUB GAD of >3-month duration	4.2% (N = 2)	3.6% (N = 3)	8% (N = 1)

*Abbreviations*: *N* Number of Studies.

The median prevalence of subthreshold GAD was generally found to rise in the general population, when the GAD time criterion was relaxed from three to one month. For subthreshold GAD lasting at least three months, the median point prevalence, 12-month prevalence and lifetime prevalence was 4.2%, 3.6% and 8% respectively. Where the condition lasted for at least one month, these figures were 5.2%, 6.1% and 12.4%. These data are all based on high-quality studies.

For specific age groups, the median of prevalences could not be determined because of N = 1 studies. A single high-quality study of young women [49] found a point-prevalence rate of 2.4% for subthreshold GAD; lower than that in the general population. By contrast, two other high-quality studies of adults/primary care patients [45,53] found higher point and lifetime prevalence rates in younger than in older people. In older adults, a single high-quality study [47] found a 12-month prevalence of 3% for subthreshold GAD and a low-quality study [48] a lifetime prevalence of 5.2%. Within the general population, older adults also seemed to have a higher 12-month prevalence rate for subthreshold GAD than other age groups [46].

For primary care patients, point prevalence rates were reported in five high-quality studies [53,55,57,58,60] and one low-quality one [50]. Although subthreshold GAD was defined variously in these studies, they suggest a median point prevalence of 5.9% (range 1.3% - 8.3%). Further discrimination, based on the duration of participants’ conditions, was not possible. In addition, one high-quality study in primary care patients [57] found a 12-month prevalence rate of 10.9% for subthreshold GAD. Beside depression, (sub)threshold GAD was cited as the most frequent mental health disorder in primary care [55]. When all cases with at least one of the core symptoms of GAD were included in this review, the median point prevalence of subthreshold GAD rose to 22.9% [50,60].

Women had higher prevalence rates for subthreshold GAD than men, independent of studies’ populations [45,46,48,55,57,60]. No systematic differences were linked to studies’ country of origin. Prevalence rates for subthreshold GAD were generally twice as high as those for threshold GAD throughout [43,46,48,51,53,56]; a picture that did not change when the single low-quality study [48] was excluded from this aspect of the analysis.

#### ***Risk, onset and course***

Subthreshold GAD was reported as a risk factor or precursor for the onset of threshold GAD [50,55]. It also raised the risk of experiencing other anxiety, mood and substance disorders. This risk was cited as substantively lower than that of threshold GAD in only one of fourteen comparisons made [56]. Subthreshold GAD was also seen to worsen the course of concurrent somatic diseases [50]. In terms of onset, course and persistence, subthreshold GAD and threshold GAD were reported as not significantly different [43,51,60]. Most subthreshold GAD cases recurred over time [51]. Whilst all but one [50] of these findings come from high-quality studies, most are of cross-sectional nature, except one [43], necessarily restricting a longitudinal view.

#### ***Comorbidity***

The rates of comorbid mental disorders were generally high in people with subthreshold GAD; similar to those occurring in threshold GAD [46,47,51,56]. More than two fifths (42%) of young women with subthreshold GAD also cited symptoms of other (sub)threshold mental health disorders [49]. Adults and primary care patients with subthreshold GAD had comorbidity rates of 86.3% and 48.5% respectively [53,56]. Various forms of anxiety disorder were most often comorbid with subthreshold GAD, followed by minor or major depression and somatoform disorders [46,47,56]. Having other comorbid mental health disorders also led to higher levels of functional impairment than having subthreshold GAD alone [52,58]. All of the above data come from high quality studies. Another high-quality study found strong links between participants’ pain disorders and subthreshold GAD, while associations between chronic pain and other subthreshold anxiety disorders were weaker [44]. In this study, participants with chronic pain and subthreshold GAD reported significantly lower levels of mental quality of life, more disability days, and greater healthcare utilization than groups without additional subthreshold GAD [44]. The results were independent of participants’ levels of comorbid depression.

#### ***Human burden***

Impairment in people with subthreshold GAD is not explained exclusively by comorbidity. Where participants had subthreshold GAD alone, or study statistics were adjusted for comorbidity, significantly higher levels of distress [43,50,55,59] and lower levels of psychosocial functioning in daily activities [43,47,49,51,58,59] occurred in all study populations – compared to groups without any symptoms of GAD. Distress or functional impairment (a key criterion of a DSM-IV GAD diagnosis) was also reported by at least 83.7% of younger (18–34 years) and 75% of older (35–65 years) participants with subthreshold GAD [46]. Subthreshold GAD cases also noted significantly greater sleep disturbance and fatigue [50,59], suicide attempts [43] and poorer perceived physical health, along with more somatic diagnoses, than controls [47,50,55,58]. In older men, subthreshold GAD was significantly linked to global cognitive impairment [54]. All of these studies, except two [50,59], contained high-quality data.

Moreover, high-quality studies showed no significant difference in the levels of distress [43,50,55] or psychosocial impairment experienced by individuals with subthreshold and threshold GAD, in any study population. Similar rates of marked social disability were found in primary care patients with chronic somatic diseases, people with threshold GAD and those with subthreshold GAD of 1–6 months duration and also less than 1 month duration [52].

However, other high quality data comparing primary care patients with subthreshold GAD to others without anxiety symptoms found no difference in psychosocial functioning, once appropriate adjustments had been made for covariates [53,55].

### Economic burden

Study results about subthreshold GAD’s economic impact were inconsistent. Two studies on the general population found no significant difference in the work performance of participants with subthreshold or threshold GAD [43,51]; impairments in both groups were significantly higher than in controls [43]. But in studies on primary care patients, the differences found between cases with subthreshold GAD and controls did not reach the level of statistical significance [50,53].

With regard to healthcare utilization and costs, primary care patients with subthreshold GAD and controls did not differ significantly on mental health visits within the previous month [53] or primary care visits within the previous three months [50]. When asked about the previous six months, patients with subthreshold GAD reported significantly more primary care visits for psychological problems than controls, independently of comorbid depression [58]. Within the last 12 months, 57.5% of participants of a general population sample with a subthreshold DSM-III-R GAD diagnosis and 50% of those with a subthreshold DSM-IV GAD diagnosis were treated by doctors or psychologists for GAD [43]. A significantly higher percentage of older adults with subthreshold GAD had a history of psychotherapy (72.7% vs. 43.8%), psychotropic medication use (81.8% vs. 28.1%) or current antidepressant or benzodiazepine use than controls (54.5% vs. 6.3%) [59]. Another study of older adults found that benzodiazepine use did not differ significantly between those with subthreshold or threshold GAD, but was significantly higher than in controls [47]. By contrast, adjusted statistics showed no more significance in the prescription of anxiolytics and antidepressants to primary care patients with or without subthreshold GAD [58]. The data from two of these studies [50,59] should, however, be interpreted with care as being of low quality.

### Prevention and therapy

The reviewed studies generally stated the importance of early detection, intervention and prevention for averting subthreshold GAD’s progression [47,50,51,55]. Whilst individuals with subthreshold GAD sought care for psychological problems significantly more often than controls [58], issues of prevention, therapy or primary care physicians’ ability to identify clinically-relevant (sub)threshold GAD were not considered. In the reviewed studies physicians identified only 39% of those with subthreshold GAD and 33% of those with threshold GAD as having clinically-relevant symptoms [58]. The reviewed studies did not recommend using current treatments for people with threshold GAD for those with GAD symptoms under the threshold for standardized diagnosis, until clinical validation in subthreshold samples [55].

## Discussion

This review found consistent evidence for high prevalence rates of subthreshold GAD; mostly twice as high as those for DSM-IV GAD. If the duration criterion for diagnosing threshold GAD is relaxed, the prevalence of subthreshold GAD increases. Subthreshold GAD is more prevalent in primary care patients than in the general population, suggesting that those affected are frequent primary healthcare resource users. In general, women are more affected by subthreshold GAD than men, as with threshold GAD [24,25]. Other populations presenting higher prevalence rates of subthreshold GAD include adolescents and older adults in contrast to middle aged individuals. Having subthreshold GAD was identified as a significant risk factor for developing threshold GAD and also elevates the risk of developing other anxiety, mood and substance use disorders. Although results show that both threshold and subthreshold GAD are generally recurrent, the latter may fail to meet the DSM-IV or ICD-10 thresholds, due to periods of symptom-free recovery. High levels of comorbidity between subthreshold GAD and other subthreshold mental health disorders, which were found throughout all study populations, may also cause this diagnostic threshold to be missed. Study results show that boundaries between different GAD symptoms are essentially arbitrary; that symptoms actually merge into one another, with significant distress and disability even in the absence of a threshold diagnosis.

Subthreshold GAD is related closely to other mental health conditions and impacts negatively on pain-related disorders. Its burden, however, cannot be explained by comorbidity alone. Results of this review show that subthreshold and threshold GAD cases of all study populations essentially experience the same degree of distress and psychosocial impairment; levels that significantly exceed those in non-anxious individuals. Only in primary care patients, study results are contradictory, also finding cases with subthreshold GAD that were no more functionally impaired than controls. In terms of work productivity, healthcare utilization and economic costs, the evidence is inconsistent. Whilst the economic burden of subthreshold GAD appears to be relatively slight, compared to the human burden, a number of high-quality studies show marked differences between those affected and controls. This is especially true for primary health care and benzodiazepine use. Comorbid subthreshold GAD can also worsen the course of other mental health and somatic disorders, further raising healthcare utilization and costs.

These results are in line with other systematic reviews of subthreshold anxiety and affective disorders. A review of the literature on panic disorders concluded that people with subthreshold panic experienced high comorbidity with other mental health disorders, as well as substantial distress and functional impairment [14]. Two more recent studies substantiated these results, finding subthreshold panic to be more prevalent than threshold panic. Subthreshold panic also raised the odds for a range of comorbid disorders and functional impairments, and increased the likelihood of healthcare utilization beyond controls [12,18]. Subthreshold social and specific phobias showed increased prevalence rates, with those affected having a higher use of benzodiazepine medications than non-anxious individuals [15,16]. The impact of subthreshold depression was also largely comparable to that of the subthreshold anxiety disorders described [13,17].

The benefit of early intervention and prevention was highly valued for individuals with subthreshold GAD and other subthreshold psychiatric syndromes [16,67,68]. Clinical trials examining people with various types of subthreshold anxiety confirm this benefit. They found preliminary evidence for the benefits of herbal medicine (lavender) compared to placebo on self-report measures for anxiety [69] and for a self-help intervention program compared to usual care, which reduced the incidence of new full-syndrome anxiety diagnoses by 50% and therefore saved health-care costs each disorder-free year [70,71].

Evidence is needed to show whether the pharmacological and counseling strategies used to treat threshold mental health conditions also benefit subthreshold ones, if they are to be prescribed [72-74]. To save healthcare resources, studies propose stepwise treatment algorithms of increasingly intensive interventions for subthreshold conditions; starting with ‘watchful waiting’ and self-help strategies (life-style changes, appropriate self-medication); working through to primary care and specialist care when symptoms persist or increase [73].

The results of this review should be interpreted in the light of certain limitations. Firstly, the literature search included only studies indexed by the stated databases, excluding non-electronic information sources. The completeness of the extracted information is therefore arguable. The absence of a unified definition of subthreshold GAD, below the standardized diagnosis, complicated the search. A myriad of possible search terms, including subthreshold, subclinical, subsyndromal, minor, partial, brief, intermittent, short-term reflects the complexity of the concept. This may have led to further relevant studies being missed. Secondly, the median prevalence estimates presented are based on an insufficient number of studies found for some subpopulations, heterogenic definitions of subthreshold GAD and inadequate response rates in more than half of the identified studies. The system used to score the reviewed papers introduces a third limitation. No consensus currently exists about the most appropriate criteria for assessing the quality of epidemiological trials. In the current review, different evidence selection criteria may have led to other quality assessments and possibly to different conclusions. Under the chosen scoring system, quality issues did not appear to influence the identified study outcomes systematically. When low-quality studies were excluded from the analysis, the evidence for subthreshold GAD’s influence did not change.

Although the impact of subthreshold GAD cannot be denied, such anxiety symptoms should not seek to be discussed in a way to lower diagnostic thresholds – for the reason that thresholds for GAD did not change essentially from DSM-IV [4] to DSM-V [75]. Further research, however, should try to clarify the thresholds between subthreshold GAD and non-pathological anxiety states. As the absence of mental health signs is not enough to ensure differentiation, with the attendant risk of over-diagnosis, the main diagnostic criterion should be significant impairment [22]. Such impairment should meet the clinical criteria for morbidity [73], including episodes of suprathreshold anxiety and effects on individuals’ work performance, social relationships and/or quality of life. It should also meet the Global Assessment of Functioning Scale’s criteria for treatment [76]. Finally, research should examine cost-effective treatment options to avert over-medicalization below the threshold of full-syndrome anxiety disorders, and to prevent progression to states of severe illness or secondary complications such as alcohol or drug misuse.

## Conclusion

Subthreshold GAD is a common, recurrent mental health disorder that causes distress and impairs psychosocial and work functioning as often as several chronic somatic diseases. In those affected, subthreshold GAD increases primary health care and benzodiazepine use. Subthreshold GAD has high comorbidity rates with other anxiety and mood disorders, somatoform and chronic pain disorders; further increasing costs.

## Competing interests

The authors declare that they have no competing interests.

## Authors’ contributions

HH has been responsible for conception, design, analysis and interpretation of data and for drafting the manuscript. HC has been involved in conception, design, analysis and interpretation of data; and in revising the manuscript critically. RL has been involved in conception, design, analysis and interpretation of data; and in revising the manuscript critically. FG has been involved in analysis and interpretation of data. GJD has been responsible for conception and design; and has been involved in revising the manuscript critically. All authors approved the final manuscript.

## Pre-publication history

The pre-publication history for this paper can be accessed here:

http://www.biomedcentral.com/1471-244X/14/128/prepub
